# Dissociation of intake and incentive sensitization during intermittent- and continuous-access heroin self-administration in rats

**DOI:** 10.1007/s00213-025-06762-6

**Published:** 2025-02-21

**Authors:** Elizabeth A. Rakowski, Christopher P. King, Brady M. Thompson, Gabriel Santos, Esther Holmes, Leah C. Solberg Woods, Oksana Polesskaya, Abraham A. Palmer, Paul J. Meyer

**Affiliations:** 1 Department of Psychology, University at Buffalo, Buffalo, NY 14260 USA; 2 Department of Internal Medicine, Wake Forest School of Medicine, Winston-Salem, NC 27109 USA; 3 Department of Psychiatry, University of California San Diego, La Jolla, CA 92093 USA; 4 Institute for Genomic Medicine, University of California San Diego, La Jolla, CA 92093 USA

**Keywords:** Opioids, Intermittent-access, Incentive sensitization, Behavioral economics, Reinstatement

## Abstract

**Rationale:**

Opioid misuse is a prominent public health concern, although patterns of use may confer different vulnerability to relapse. Continuous-access (**ContA**) self-administration has traditionally been used in preclinical models to study drug-motivated behaviors and produces robust escalation of intake and tolerance development. Alternatively, studies using intermittent access (**IntA**), where self-administration occurs in discrete drug-available periods, suggest that overall intake may be dissociable from subsequent increases in motivation (i.e., incentive sensitization). However, IntA paradigms have focused primarily on psychostimulants like cocaine and methamphetamine and have not been as comprehensively studied with opioids.

**Objective:**

We compared two paradigms of heroin self-administration, ContA and IntA, to assess their effect on heroin intake and motivation.

**Methods:**

Male and female rats were trained to self-administer heroin, then were transitioned to either ContA or IntA paradigms. Following self-administration, rats were tested in progressive-ratio, behavioral economics threshold probe, and conditioned reinforcement tests to measure motivation-related behaviors.

**Results:**

Both patterns of intake evoked similar heroin-directed motivation during progressive-ratio and conditioned reinforcement tests, despite lower overall intake throughout IntA for male rats. Females had similar responding between treatments in self-administration and progressive-ratio even though IntA rats had less time to earn infusions. During threshold probe, IntA-trained subjects showed more inelastic responding (lower α values), suggesting a greater degree of dependence-like behavior.

**Conclusions:**

These results suggest the importance of dissociating heroin intake from incentive sensitization and emphasize the significance of sex differences as a modifier of heroin consumption and motivation.

## Introduction

Opioid use disorder (**OUD**) is an epidemic in the United States that has been augmented by the COVID-19 pandemic and is the major cause of opioid-related deaths (Hedegaard et al.^2021^; Wang et al. 2023). Nearly one in five overdose-related deaths in the United States involved heroin in 2020, quadrupling the rate of heroin-involved fatalities since 2010 (Centers for Disease Control and Prevention 2022), and approximately 5.6 million Americans had an OUD diagnosis in 2021 (Substance Abuse and Mental Health Services Administration 2022). One of the key features of OUD is intense motivation to find and secure supply of the drug (American Psychiatric Association 2013), which preclinical models measure by increasing the response requirement to receive drug access or decreasing the amount of the drug that is given for each response. Currently, several studies have investigated heroin-related motivation, but no studies that have considered how the pattern of drug self-administration affects subsequent motivation as measured by changes in response requirement.

Experimental paradigms used in rodents demonstrate that continuous-access (**ContA**) heroin self-administration sessions for extended periods of time lead to escalation of intake and increased responding during reinstatement, two components of OUD. For example, six-hour sessions resulted in escalated heroin intake, increased resistance to extinction, and increased responding during, cue-, stress- and heroin-induced reinstatement sessions (Ahmed et al. 2000; Lenoir & Ahmed 2007; Zhang et al. 2004). However, heroin intake does not always lead to enhanced motivation to work for heroin (i.e., incentive sensitization; Robinson & Berridge 1993). For example, in a study in which rats self-administered escalating doses of heroin, there was no subsequent increase in responding during a progressive-ratio test (Minhas & Leri 2014).

Overall heroin intake is only one factor influencing incentive sensitization. Another factor is the pattern of self-administration. Compared to rats with continuous access to cocaine and fentanyl, rats with intermittent access (**IntA**) displayed more incentive sensitization, as measured during progressive-ratio and reinstatement tests (Fragale et al. 2021; Zimmer et al. 2012). This occurred despite IntA producing less total intake than ContA paradigms (Allain et al. 2018; Allain & Samaha 2019; Kawa et al. 2016). IntA models the intermittent nature of drug intake in humans, in which limited drug availability can lead to intermittent, ‘binge’-like drug self-administration, during which other risky behaviors are more likely (Cohen and Sas 1994; Roy et al. 2017; Ward et al. 1997; Beveridge et al. 2012), but this has not been as extensively studied for opioids. In one study, rats that self-administered heroin on an IntA schedule had higher overall intake during self-administration and responded more for the associated cue-light during a reinstatement test, compared to rats self-administering heroin on a ContA schedule (D’Ottavio et al. 2022). Those results indicated the importance of self-administration pattern in animal models of relapse. However, the effect of self-administration pattern (ContA vs. IntA) on other measures of heroin motivation have not been studied.

The present study aimed to address this by assessing the effects of the IntA model on heroin incentive sensitization in male and female heterogeneous stock (**HS**) rats, utilizing measures of heroin demand, including progressive-ratio, behavioral economics, and cue-reinstatement testing. A strength of this paradigm is the ability to test behaviors throughout the IntA or ContA experience, such as administering a progressive-ratio test of motivation before and after the self-administration phase, to track the effects of increasing drug exposure on various behaviors on an individual level via within-subjects analysis. We hypothesized that the IntA paradigm would induce higher responding for heroin (D’Ottavio et al. 2022), specifically during progressive-ratio and behavioral economics testing. We also predicted that there would be no sex differences in responding, based on the lack of differences between females and males in total heroin intake or frequency of infusions previously reported (D’Ottavio et al. 2022).

## Methods & materials

### *Animals & housing*

**Fig. 1:**
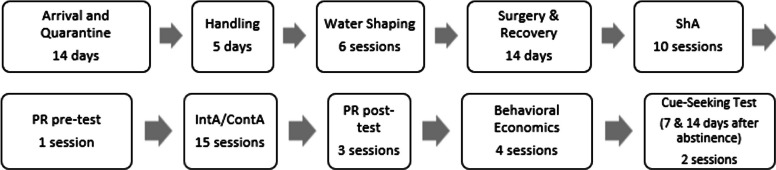
General timeline of experimental procedures. ShA = short access/acquisition phase; PR = progressive-ratio; IntA/ContA = intermittent-access/continuous-access

**65** adult female (*n* = **28**) and male (*n* = **37**) HS rats [NMcwiWFsm:HS rats (RRID:RGD_13673907); Solberg Woods et al. 2010] were provided by Dr. Leah Solberg Woods and shipped from Wake Forest University School of Medicine (Winston-Salem, NC) from 2021–2022, weighing on average 224 (175–272) grams for females and 368 (309–467) grams for males. Upon arrival at University at Buffalo, rats were pair-housed on a 12:12-h reverse light cycle in polycarbonate cages in temperature- and humidity-controlled rooms. Rats had ad libitum access to food and water unless specified in behavioral procedures. They were quarantined for two weeks, then allowed two days to acclimate to the facility before daily handling by the experimenter was performed for five days. All procedures were approved by the University at Buffalo Institutional Animal Care and Use Committee and were carried out in accordance with all relevant guidelines and regulations (National Research Council 2011). A general timeline of the experiment can be found in Fig. 1.

### Drugs

Diacetylmorphine hydrochloride was provided by the National Institute on Drug Abuse’s Drug Supply Program (catalog #9200–001) and diluted in 0.9% sterile saline to make a 0.0847 mg/mL solution. This concentration was chosen so the infusion pump would run for approximately (adjusting for rats’ weight) four seconds to deliver a 0.02 mg/kg intravenous (IV) infusion. Similarly, saline was used to dilute the: heparin solution (30 units/mL; Laboratory Animal Facility; University at Buffalo, NY), ketamine IV flush solution (10 mg/mL; Patterson Veterinary; Mt. Joy, PA), and carprofen solution administered subcutaneously (5 mg/mL; Patterson Veterinary; Mt. Joy, PA). Enrofloxacin (22.7 mg/mL; Patterson Veterinary; Mt. Joy, PA) was co-administered IV with the heparin (30 units/mL) flush solution after surgery.

### Surgical procedures

Rats were implanted with chronically indwelling catheters in the right jugular vein while under isoflurane anesthesia (1–3%). A 14-cm catheter line with bead at 3-cm (RJVR-50; SAI Infusion Technologies; Lake Villa, IL) was inserted into the jugular vein, secured with suture (3–0 USP, Avantor; Radnor, PA), and connected subcutaneously to an externalized vascular access button (VABR1B/22, Instech Labs; Plymouth Meeting, PA) located posterior to the scapular region on the dorsal side of the rat. Post-operative care included a daily subcutaneous injection of carprofen (5 mg/kg) for two days for pain management, and a daily flush of heparinized saline (30 units/mL) and enrofloxacin (10 mg/kg) for 10 days following catheter implant to prevent infection and maintain catheter patency. Catheter patency was verified before the beginning of the experiment and once per week on a non-testing day for the duration of the experiment by flushing up to 0.05 mL of ketamine (10 mg/mL) into the catheter and observing for muscular ataxia. Rats that failed this test were removed from the study.

### Behavioral procedures

*Water-shaping*. This procedure used brief water restriction to promote lever-pressing behavior before heroin self-administration began. Rats’ water bottles were removed from their home cages for 23 h before being placed in standard operant chambers (Med Associates Inc.; St. Albans, VT) located in noise- and light-attenuating boxes for one hour per day for up to six days. For the first two days, levers remained retracted and stimulus lights remained off. Rats were given 30-s access to a sipper bottle filled with tap water every 60 s. For the next four days, the levers were presented in the chamber, and a single press of the active lever (counterbalanced for left/right position) resulted in retraction of both levers and sipper bottle access for 15 s. Rats were considered to have acquired lever-pressing when there were at least 15 active lever presses in one hour, after which, water was returned to their home cages for the remainder of the experiment. Once this criterion was met, rats were not tested the rest of the water-shaping phase to ensure that animals were not over-trained once the skill was acquired. Rats that did not meet the minimum lever press requirement received 10 min of access to water in their home cage then were water-deprived until the next day’s test. All rats continued to the heroin acquisition phase of the experiment regardless of if they acquired lever pressing. This decision was made due to our previous experience that most animals that did not acquire the skill during water-shaping will learn to lever-press when the reinforcer is changed to a drug (i.e. heroin, cocaine) and we wanted to keep exposure to this phase and to the self-administration chamber similar for all animals. 30 of 65 rats met the lever-press requirement during water-shaping. Of the 35 rats that failed to meet criterion during water-shaping, 25 rats later acquired the lever-pressing response during the short-access heroin acquisition phase. Eight rats did not acquire lever-pressing by the end of this short-access phase and were not tested further or included in statistical analyses. Two rats were also lost during surgery after water-shaping. 55 rats entered the short-access self-administration phase.

*Intravenous self-administration.* All self-administration during this phase was on a fixed-ratio one (FR1) schedule of reinforcement. Rats were given a Nyla-bone in the chamber to prevent opioid-induced chewing of the catheter tether and forelimbs, which has been observed in previous studies (Chen et al. 2006; Kenny et al. 2006). Rats were first tested with daily two-hour acquisition sessions (“short-access” self-administration) conducted across 10 days. For each session, the vascular access buttons were connected to an infusion line housed in a tether (VABR1T/22) encased in a flexible metal spring to minimize chewing, which was connected to a one channel swivel (375/22PS) connected to a drug delivery arm (PHM-110-SAI; Instech Laboratories Inc.; Plymouth Meeting, PA) to enable unrestricted movement. In the operant chamber, there was a red house light, two retractable levers, and a light above each lever. Each active-lever press was reinforced with a heroin infusion which resulted in the retraction of both levers, display of the light above the active lever for eight seconds, and a 0.02 mg/kg heroin infusion [dose based on findings from Dai et al. (1989)] delivered via a syringe pump located outside the chamber. Once this process was completed, the lever light turned off and both levers were presented back into the chamber. Inactive-lever presses were recorded but had no consequence.

52 rats (attrition from short-access = 3) continued to the next self-administration phase and were assigned into either the ContA or IntA group; the groups were matched based on total intake on the last session of the short-access phase and progressive ratio pre-test responding. There were 15 daily four-hour sessions, where ContA rats had uninterrupted access to heroin infusions and IntA rats had eight five-minute drug-available periods separated by 25-min unavailable periods for a total four hours. During the unavailable periods, the levers were retracted and chamber light was illuminated. Measures taken during self-administration included number of infusions earned, active and inactive lever presses, and locomotor data as determined by locomotor beam breaks. Inter-infusion intervals were calculated to determine the range of time occurring between infusions, by subtracting the timestamps of each infusion from the next occurring infusion and categorizing those by minute.

*Progressive-ratio.* 49 rats (attrition from self-administration = 3) underwent progressive-ratio tests before (one test) and after (three tests) the 15 days of ContA or IntA self-administration. During these tests, the response requirement for each heroin infusion increased as follows: 1, 2, 4, 6, 9, 12, 15, 20, 25, 32, 40, etc., according to the Eq. (5* *e*0.2*n*)−5 using the nearest whole number (Richardson and Roberts 1996). Breakpoint was defined as the last ratio achieved before the rat stopped responding for one hour or by the end of the four-hour session. The time at breakpoint, number of infusions earned, number of active and inactive lever presses, and number of locomotor beam breaks were also measured. Separate rats were tested with the training dose of heroin (0.02 mg/kg/infusion) and another group of rats with half of this dose (0.01 mg/kg/infusion) to determine if the breakpoints were dose-dependent.

*Threshold probe.* 42 rats (attrition from progressive-ratio = 7) were given two ContA or IntA reacquisition sessions after the progressive ratio tests to reestablish self-administration responding. Rats were then tested using a behavioral economic procedure which consisted of 130-min sessions for four days. These tests were separated over two days to reduce the accumulation of heroin’s active metabolites from the higher dose to the lower doses, but to enable multiple doses for the construction of robust demand curves. During each session, the heroin dose earned on an FR1 schedule decreased incrementally during 30-min blocks. During each block, rats received a 10-min drug available period followed by a 20-min unavailable period, which was included to allow partial clearance of heroin and its metabolites. The doses for day 1 and 3 were as follows: 35.576 µg/mL, 11.244 µg/mL, 3.553 µg/mL, 1.123 µg/mL, 0.355 µg/mL. The doses for day 2 and 4 were as follows: 20.000 µg/mL, 6.321 µg/mL, 1.998 µg/mL, 0.631 µg/mL, 0.200 µg/mL. As the dose decreases, animals must respond more to reach the desired brain level of heroin. This number of responses is considered the “price”. Three main measures of heroin demand will be calculated: (a) P_max_: maximum price an animal is willing to “pay” as response requirement, or “cost”, increases, (b) Q_0_: preferred level of reward consumption when work to acquire cost is negligible, and (c) alpha (α): slope of the calculated demand curve also known as normalized demand elasticity that reflects the essential value of the reward. Procedures adapted from Newman and Ferrario (2020) and Bentzley et al. (2013).

*Conditioned reinforcement.* 40 rats (attrition from threshold probe = 2) were given another two ContA or IntA reacquisition sessions, then remained in their home cages with experimenter-imposed abstinence to heroin for six days. On the following (7th) day, rats were placed in the operant chamber for 15 min during which active lever pressed were reinforced by the illumination of the heroin associated stimulus light. Rats remained in their home cages for another six days, then given a second test on the following (14th) day. Measures included the number of cue presentations, and number of active and inactive lever presses.

### Data analyses

For all data, the number of active lever presses, inactive lever presses, and infusions earned were analyzed using repeated measures analysis of variance (ANOVA) with *Session* as the within-subjects factor. *Sex* (male vs. female) and *Access* (ContA vs. IntA) were the between-subjects factors. Additionally, factorial ANOVAs are used to determine differences in breakpoint and time to breakpoint for the progressive-ratio tests, and P_max_, Q_0_, and α for behavioral economics. *Sex* (male vs. female) and *Access* (ContA vs. IntA) were the between-subjects factors for all factorial ANOVAs. Post-hoc analyses were conducted using Bonferroni-corrected comparisons when applicable. Statistical analyses were conducted in the Statistica 12 software package and all graphs were created using the SigmaPlot 12.5 software package.

## Results

### Self-administration

**Fig. 2:**
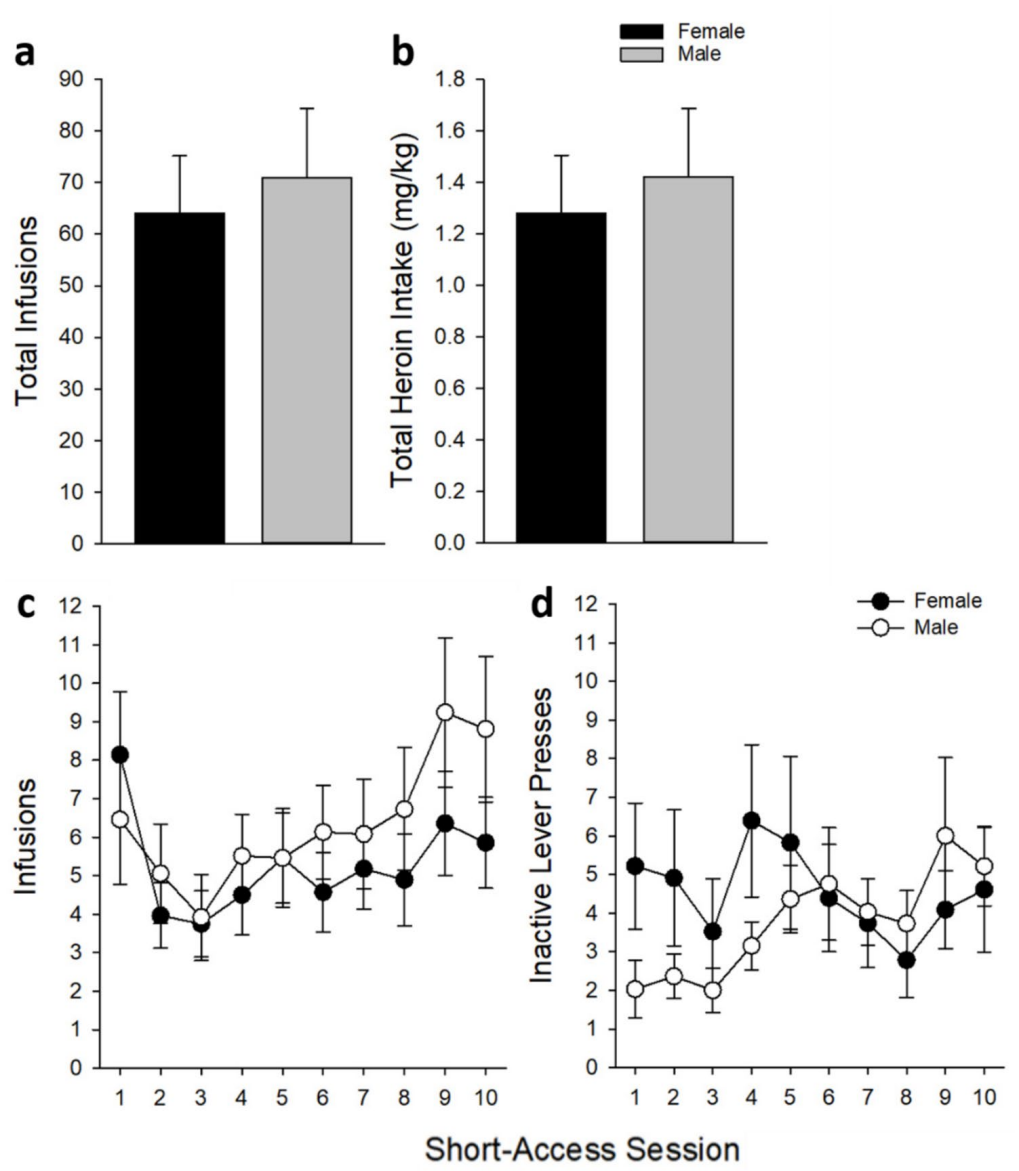
Short-access heroin acquisition phase for male (*n* = 37) and female (*n* = 28) rats. Mean (± SEM) **a** total infusions earned, **b** total heroin intake in mg/kg, **c** infusions earned by session, and **d** inactive lever responses by session are separated by *Sex*. There was a significant increase in earned infusions for all rats regardless of sex during sessions 9 and 10 compared to session 3 (*p*’s < 0.05)

During the short-access acquisition phase, rats significantly increased their heroin consumption [main effect of *Session*: *F*_(9,546)_ = 5.71, *p* < 0.001; see Fig. 2] and active lever responding [main effect of *Session*: *F*_(9,546)_ = 5.52, *p* < 0.001], such that both measures were significantly higher for sessions 9 and 10 compared to session 3 (*p’*s < 0.05). There was a non-significant trend toward higher responding in male rats during the last two sessions compared to responding in males on the third session (*p* = 0.077). There was also a significant *Session* by *Sex* interaction (*F*_(9,546)_ = 2.32, *p* < 0.05), where male but not female rats increased inactive lever-presses during session 9 when compared to the first and third sessions (*p’*s < 0.05).

**Fig. 3:**
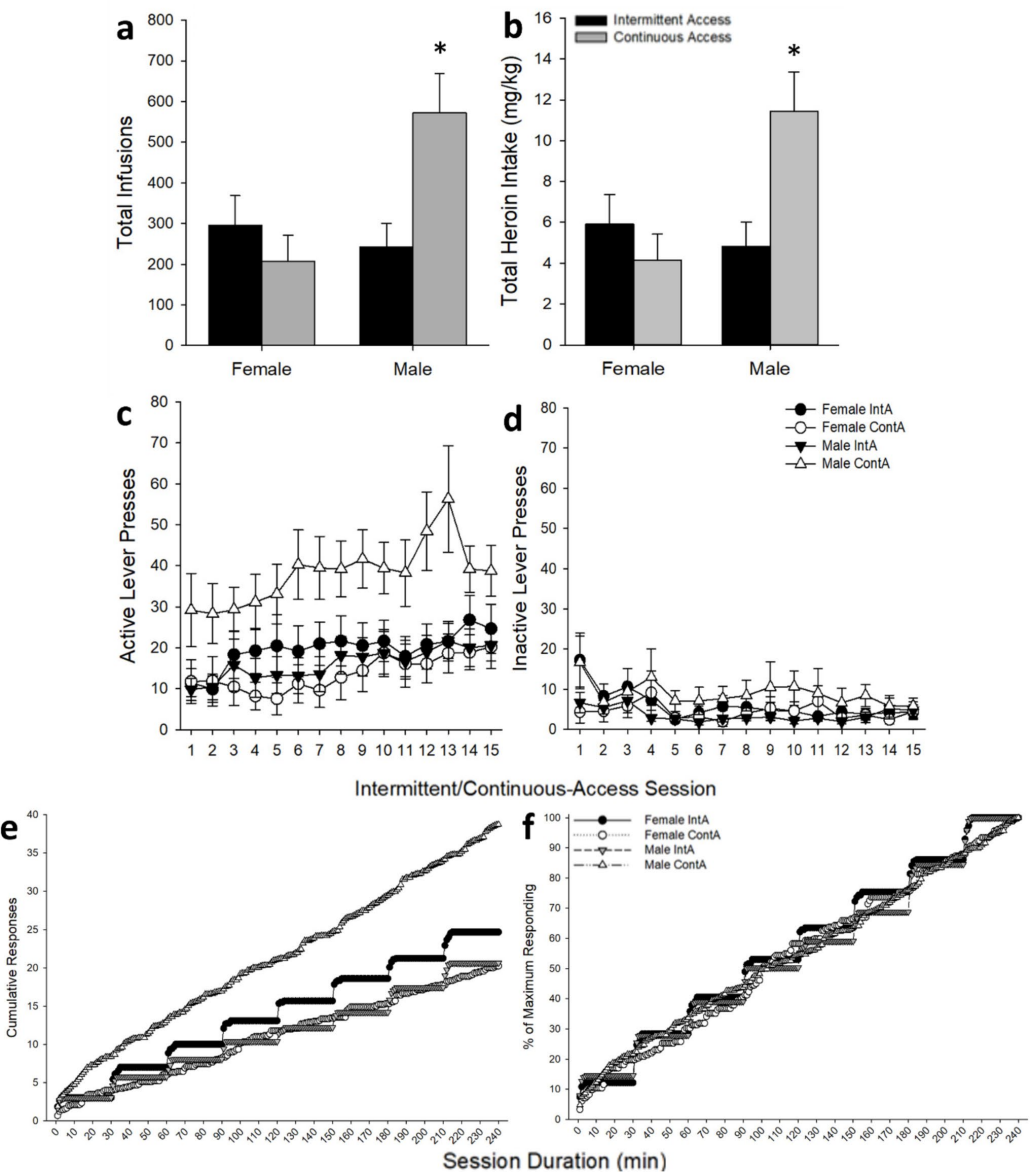
Intermittent/continuous-access heroin self-administration phase for female (*n* = 21; ContA *n* = 9, IntA *n* = 12) and male (*n* = 31; ContA *n* = 15, IntA *n* = 16) rats. Mean (± SEM) **a** total earned infusions, **b** total heroin intake in mg/kg, **c** infusions earned by session, **d** inactive lever responses, **e** cumulative responding, and **f** percent of maximum responding by minute during the last self-administration session are separated by *Sex* and *Access Group*. Asterisks (*) indicate that male rats in the continuous-access group earned more infusions and had higher total heroin intake than all other groups (*p*’s < 0.05)

**Fig. 4:**
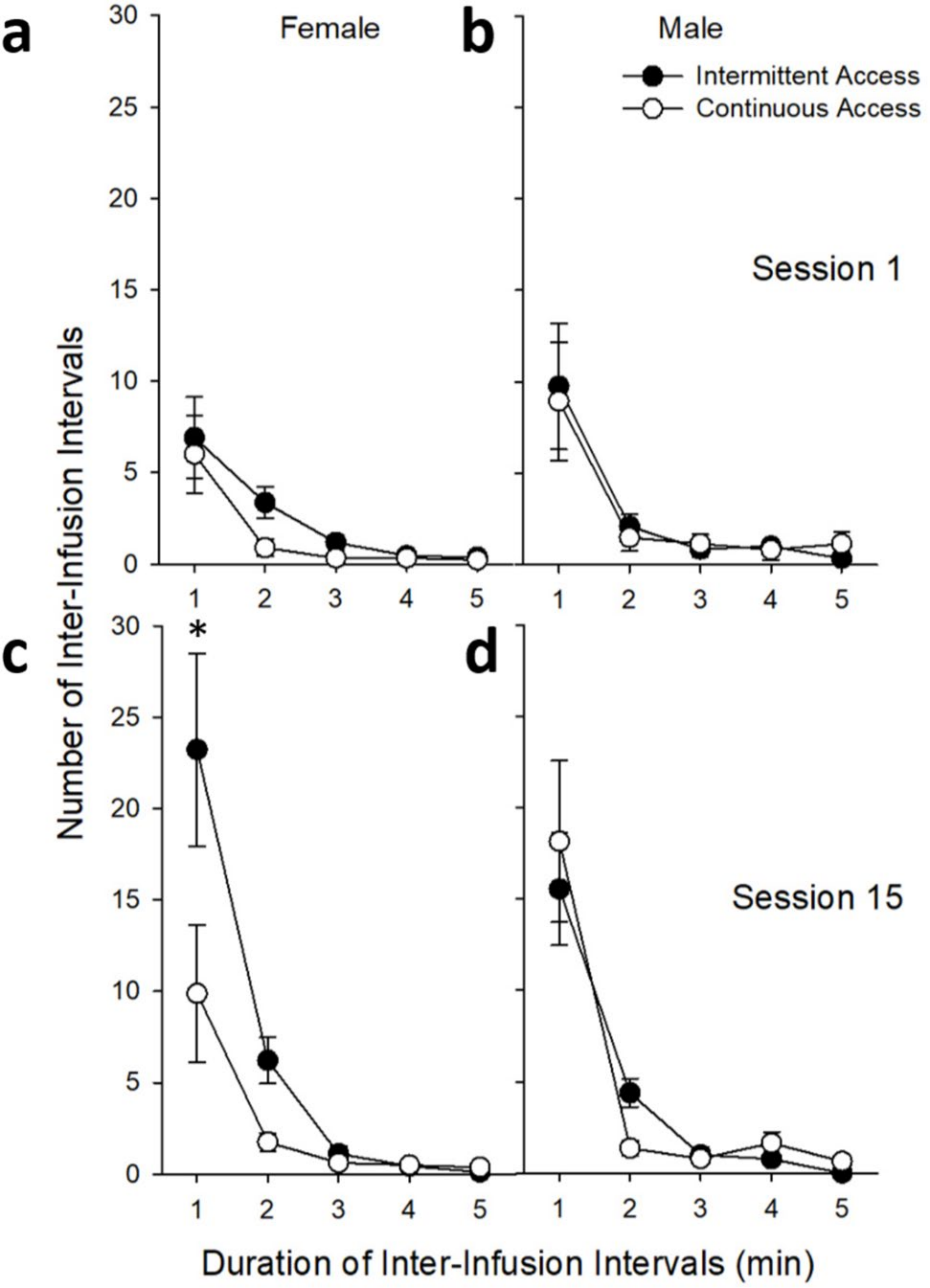
Mean (± SEM) number of inter-infusion intervals in minutes during intermittent/continuous-access heroin self-administration phase in female (*n* = 21; ContA *n* = 9, IntA *n* = 12) and male (*n* = 31; ContA *n* = 15, IntA *n* = 16) rats are separated by *Sex* and *Access Group*. **c** Asterisk (*) indicates a statistically significant difference in session 15, where intermittent-access resulted in more one-minute inter-infusion intervals than continuous-access in females (*p* < 0.001)

Following short-access, rats were assigned to either the IntA or ContA paradigms. All groups escalated heroin intake during this phase [main effect of *Session*: *F*_(14,546)_ = 5.71, *p* < 0.001], however there was also a significant *Sex* by *Access Group* interaction (*F*_(1,39)_ = 6.74, *p* < 0.05), where ContA male rats responded more than IntA male rats while no difference was seen in females (see Fig. 3). There was also a significant *Sex* by *Access Group* by *Minute* interaction for inter-infusion intervals during the last session (*F*_(1,48)_ = 6.89, *p* < 0.05), where IntA resulted in more one-minute intervals between infusions than ContA for females (*p*’s < 0.001; see Fig. 4). These results suggests that IntA females took the heroin infusions more rapidly than ContA females where infusions were taken with more time in between.

### Progressive ratio

**Fig. 5:**
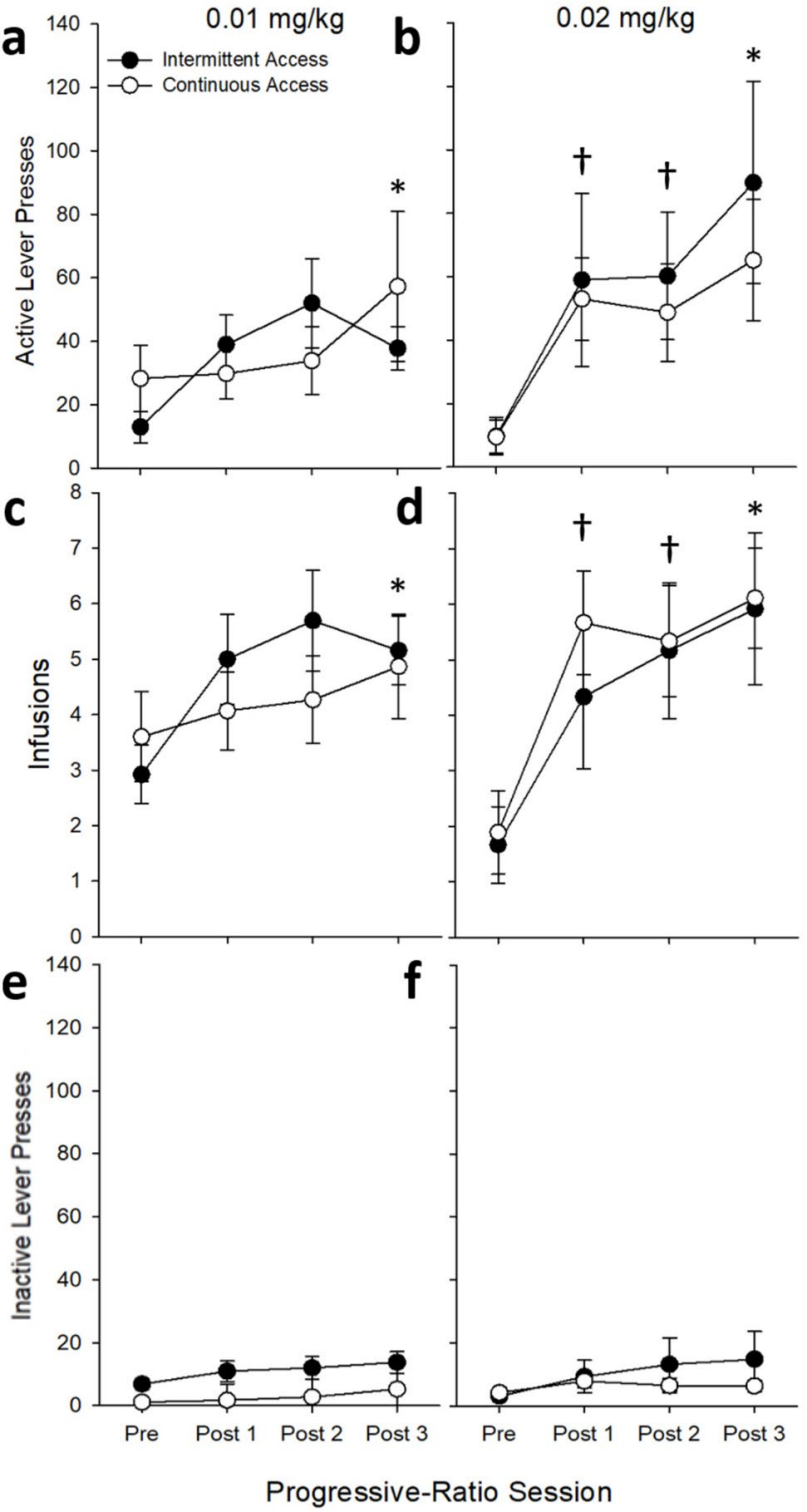
Mean (± SEM) **a, b** active lever presses, **c, d** infusions, and **e, f** inactive lever presses during progressive ratio pre- and post-tests separated by *Access Group* and *Heroin Dose* (0.01 mg/kg: ContA *n* = 15, IntA *n* = 13; 0.02 mg/kg: ContA *n* = 9, IntA *n* = 12). There were no statistically significant differences between *Sex* or *Access Group*. Asterisk (*) indicates a statistically significant main effect of *Session*, where number of infusions and active lever presses was significantly higher during the 3rd post-test compared to the pre-test for both doses (*p*’s < 0.05). Additionally, the obelisk **(†)** indicates a significant *Session* x *Dose* interaction, in which responding during the first and second post-tests was also greater than the pre-test only for 0.02 mg/kg heroin infusions

Regardless of dose (0.01 or 0.02 mg/kg), there was a main effect of *Session* on active lever responding (0.01 mg/kg: *F*_(3,72)_ = 3.36, *p* < 0.05; 0.02 mg/kg: *F*_(3,51)_ = 7.36, *p* < 0.001), earned infusions (0.01 mg/kg: *F*_(3,72)_ = 6.72, *p* < 0.001; 0.02 mg/kg: *F*_(3,51)_ = 15.94, *p* < 0.001), and breakpoint ratio (0.01 mg/kg: *F*_(3,72)_ = 3.88, *p* < 0.05; 0.02 mg/kg: *F*_(3,51)_ = 8.83, *p* < 0.001). Post-hoc analyses revealed that all measures were significantly higher during all of the post-tests than the pre-test at the 0.02 mg/kg dose, whereas only the last post-test was higher than the pretest for the 0.01 mg/kg dose (*p*’s < 0.05; See Fig. 5).

**Fig. 6:**
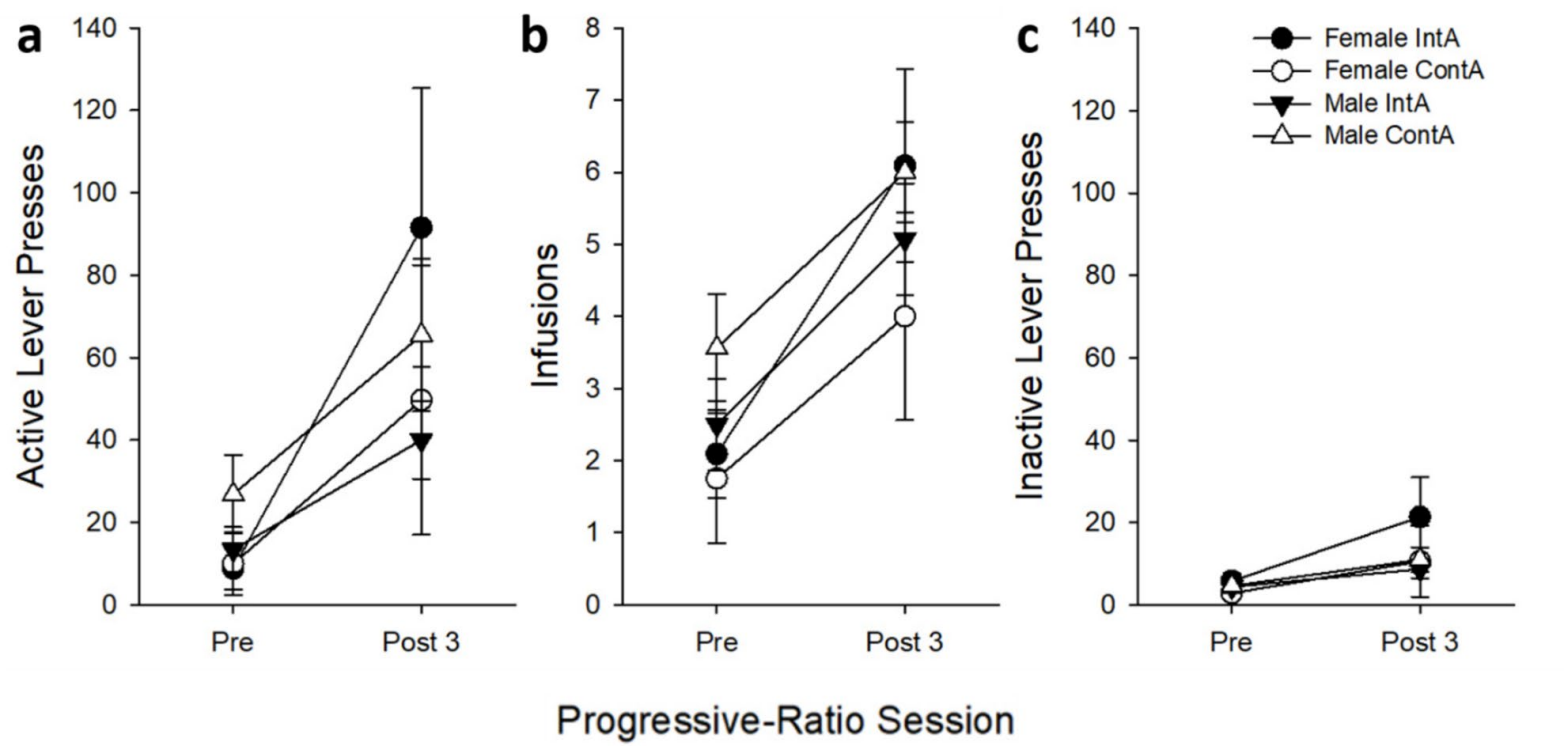
Mean (± SEM) **a** active lever presses, **b** infusions, and **c** inactive lever presses during progressive ratio pre-test and last post-test separated by *Access Group* and *Sex* (female: ContA *n* = 8, IntA *n* = 11; male: ContA *n* = 16, IntA *n* = 14)

For inactive lever responses, there was a main effect of *Session* for the 0.01 mg/kg dose (*F*_(3,72)_ = 6.69, *p* < 0.001); post-hoc analysis revealing that the last post-test had higher responding compared to the pre-test. For the 0.02 mg/kg dose, a main effect of *Session* on time to reach breakpoint was due to the post-times all being longer than the pre-test time (all *p’s* < *0.05)*. *Sex* was not analyzed due to the low number of females in the ContA group tested with the 0.02 mg/kg dose (*n* = 2) caused by attrition throughout the experiment from catheter patency loss and infection. When collapsed across heroin dose, a similar increase in active lever responding can be observed (see Fig. 6).

### Behavioral economics

**Fig. 7:**
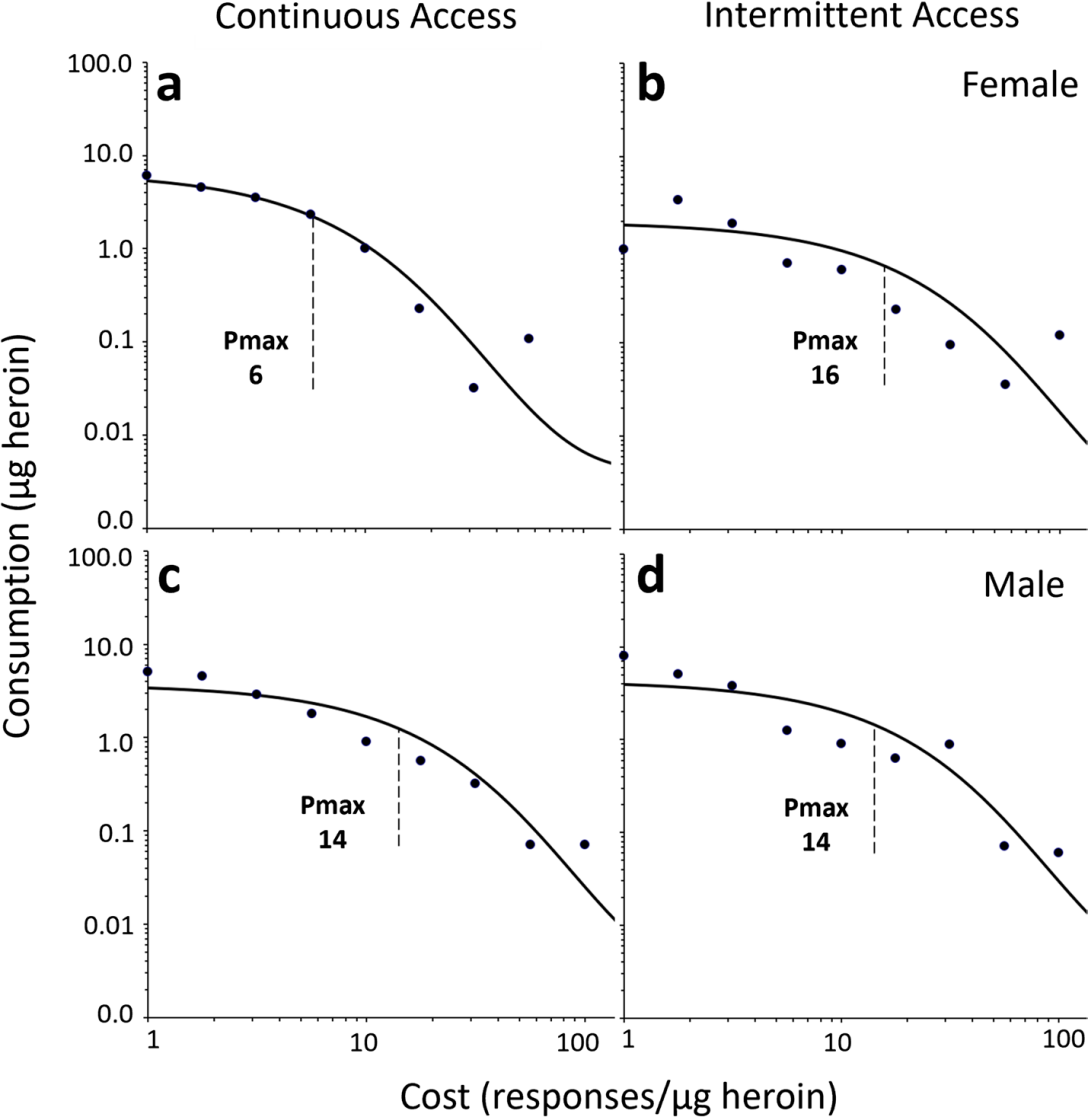
Representative demand curves of **a** female ContA, **b** female IntA, **c** male ContA, and **d** male IntA subjects

**Fig. 8:**
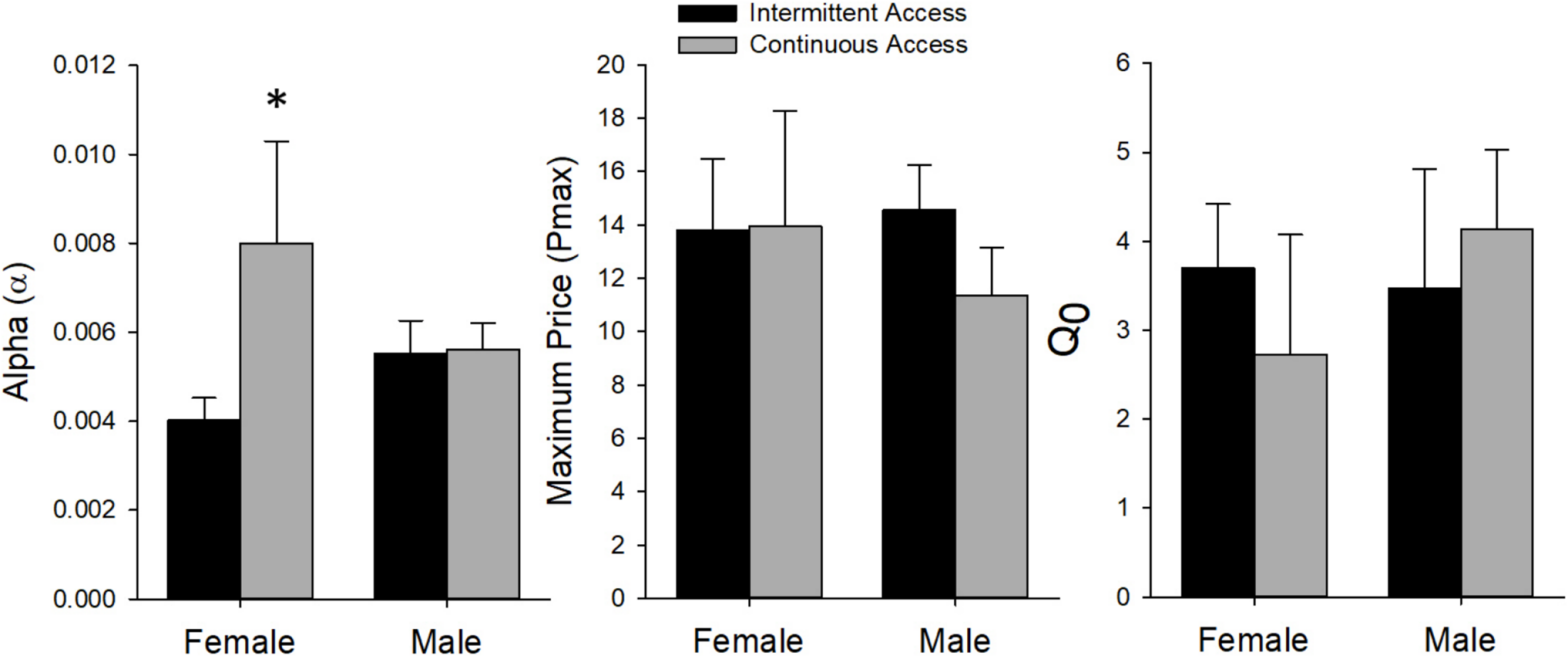
Mean (± SEM) calculated values from the behavioral economics threshold test separated by *Sex* and *Access Group*; female *n* = 15 (ContA *n* = 6, IntA *n* = 9), male *n* = 27 (ContA *n* = 14, IntA *n* = 13). Asterisk (*) indicates a statistically significant difference, where continuous access led to significantly higher Alpha values than intermittent access in females (*p* < 0.05)

**Fig. 9:**
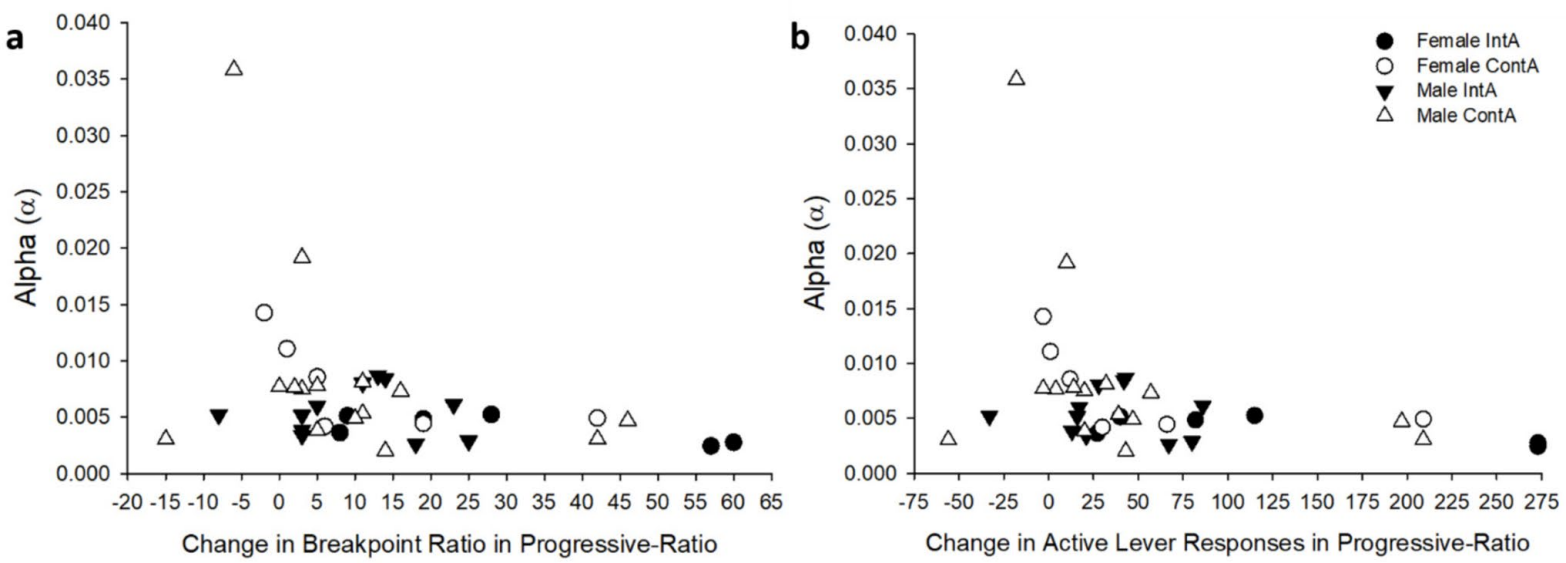
Scatter plots depicting relationship alpha values during the threshold test and change from the pre-test to last post-test during progressive-ratio in (**a**) breakpoint ratio (r = −0.36) and (**b**) active lever responding (r = −0.34), separated by *Sex* and *Access Group*

The data from the first iteration of the threshold test were analyzed, since overall responding decreased during the second test. Example behavioral economics curves using the focused-fit approach (Bentzley et al. 2013) are shown in Fig. 7; these curves were used to calculate α, P_max_, and Q_0_. There was a *Sex* by *Access Group* interaction for α (*F*_(1,29)_ = 4.22, *p* < 0.05), where post-hoc analysis revealed that ContA rats had higher values than IntA rats only for females (*p*’s < 0.05; see Fig. 8). Motivation for heroin is inversely proportional to α (Bentzley et al. 2013; Bentzley et al. 2014; Hursh and Silberberg 2008), suggesting that ContA leads to lower motivation to work for heroin in female rats, but not for males. Further, α was significantly negatively correlated with change in breakpoint ratio and active-lever responding during progressive-ratio (breakpoint ratio: r = −0.36, active lever responding: r = −0.34; see Fig. 9). In other words, rats that increased responding and breakpoint ratio from the pre-test to the last post-test of progressive-ratio had lower sensitivity to change in demand for heroin during the threshold test. There were no other significant differences between *Sex* (P_max_: *p* = 0.71; Q_0_: *p* = 0.65) or *Access Group* (P_max_: *p* = 0.55.; Q_0_: *p* = 0.91).

### Conditioned reinforcement

**Fig. 10:**
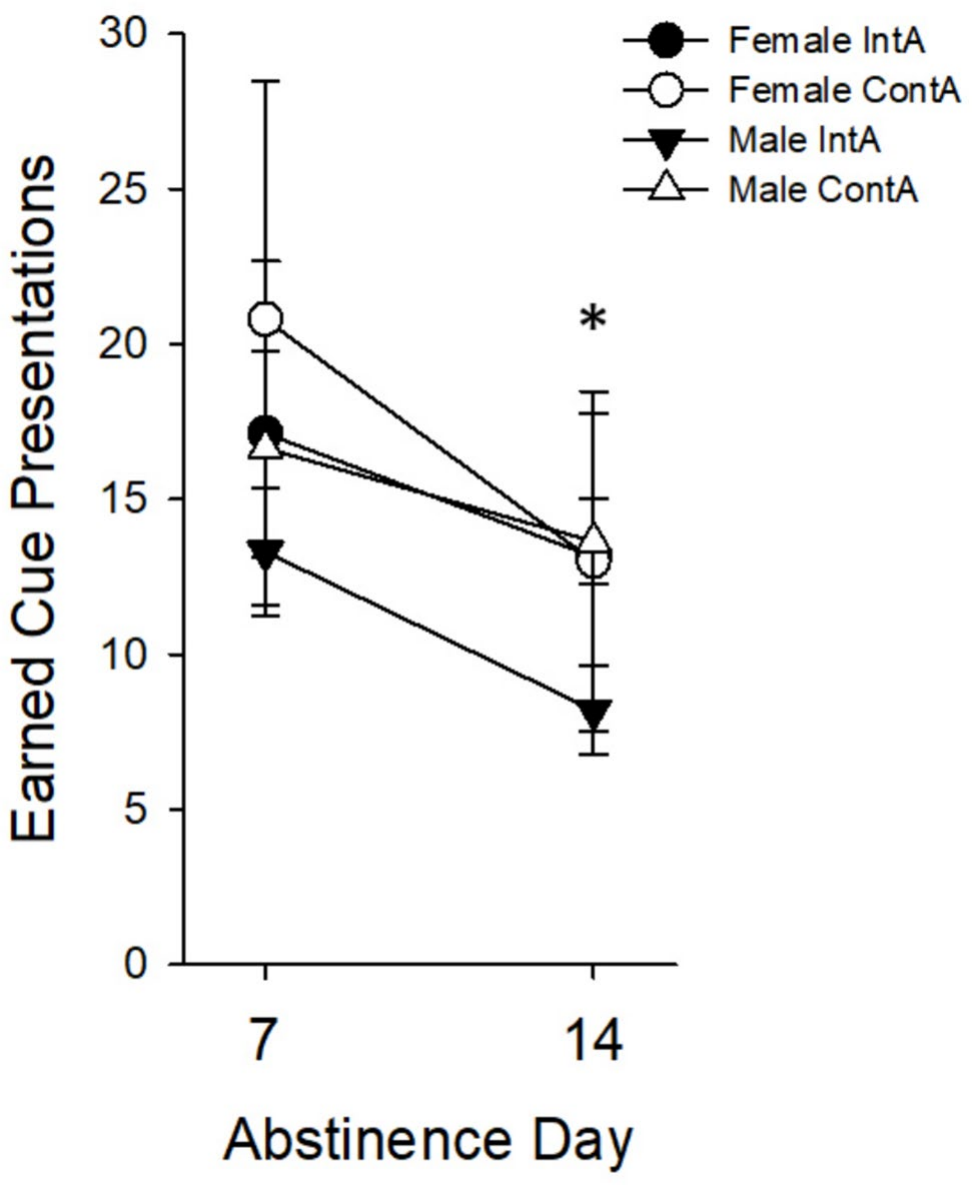
Mean (± SEM) cue presentations earned during the conditioned reinforcement tests separated by *Sex* and *Access Group*; female *n* = 13 (ContA *n* = 5, IntA *n* = 8), male *n* = 27 (ContA *n* = 14, IntA *n* = 13). Asterisk (*) depicts a significant main effect of *Session*, where cue presentations decreased on abstinence day 14 when compared to abstinence day 7 regardless of *Sex* or *Access Group* (*p* < 0.05)

Rats next underwent experimenter-imposed abstinence for 7 and 14 days, then were tested for responding for the heroin-paired cue in the absence of the drug. We found that active-lever responding decreased similarly regardless of *Sex* or *Access Group* on abstinence day 14 when compared to abstinence day 7 (*F*_(1,28)_ = 5.82, *p* < 0.05; see Fig. 10).

### Correlational analyses

Correlation coefficients were calculated to determine the relationships between total heroin infusions, one-minute inter-infusion intervals during the last self-administration session, and measures of motivation including progressive-ratio, behavioral economics, and conditioned reinforcement (i.e. cue-seeking). Cumulative heroin infusions were specifically correlated with responding for heroin when effort was low (Q0; r = 0.37) while 1-min inter-infusion intervals were correlated with responding during progressive-ratio testing (r = 0.30) in all animals. Both cumulative intake and 1-min inter-infusion intervals were correlated with cue-seeking behavior during the conditioned reinforcement tests (r’s = 0.38–0.44; see Table 1).

**Table 1.**
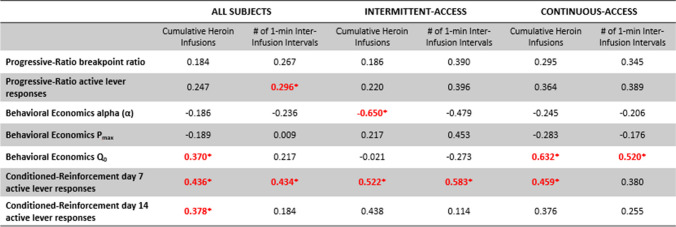
Correlation coefficients showing the relationships between cumulative heroin infusions and one-minute inter-infusion intervals on various behaviors measured during the progressive-ratio, behavioral economics, and conditioned reinforcement, separated by all subjects, intermittent-access only, and continuous-access only

Highlighted coefficients with an asterisk (*) indicate statistically significant correlations

When these analyses were separated by access group, different patterns emerged. The two correlations found between progressive-ratio responding and 1-min inter-infusion intervals and cumulative heroin infusions and responding during conditioned reinforcement on abstinence day 14 did not remain, possibly due to lack of statistical power or large standard deviation values. The correlation with Q0 maintained only in continuous-access rats (r = 0.63) along with an additional correlation between Q0 and 1-min inter-infusion intervals (r = 0.52). The correlations between responding during conditioned reinforcement on abstinence day 7 with both cumulative heroin infusions and 1-min inter-infusion intervals remained for intermittent-access animals, but only the correlation with cumulative heroin infusions carried over for continuous-access animals. The last correlation to note between α values in behavioral economics and cumulative heroin infusions appeared only in intermittent-access animals (r = −0.65).

## Discussion

Overall, we found that the effects of total drug exposure were partially dissociated from increases in motivation (i.e., incentive sensitization), indicating that the pattern of drug access is an additional factor that can impact subsequent heroin motivation along with overall intake, as has been seen in previous studies with cocaine (Beasley et al. 2023; Kawa et al. 2016; Zimmer et al. 2012). Despite higher intake in ContA males, IntA males showed comparable levels of motivation as seen by similar increases in responding during progressive-ratio, similar maximum price scores calculated from the behavioral economics threshold test, and responding for cues during conditioned reinforcement. However, without a 40-min ContA comparison group, it is unclear the degree to which 4-h ContA and IntA increased drug motivation in comparison to such a group. In females, there was similar intake between IntA and ContA groups, but IntA led to lower demand elasticity during the behavioral economics threshold test as indicated by α values. This indicates a possible dissociation between total drug intake and incentive motivation, where these behaviors have the potential to independently affect motivational behaviors. Lower inelasticity (α) values in females during the behavioral economics threshold procedure suggests that IntA produced sustained responding across decreasing heroin doses compared to rats exposed to ContA.

These differences are accompanied by a qualitatively different pattern of intake during IntA compared to ContA. IntA led to more one-minute inter-infusion intervals by the last self-administration session compared to ContA and there is a step-wise pattern to intake rather than a steady cumulative trend as seen in ContA. This result indicates that IntA may be mimicking binge-like drug-taking, which has been associated in past studies with higher brain concentrations of drug and higher motivation for drugs of abuse (Bouayad-Gervais et al. 2014; Zimmer et al. 2012). Further, correlational analyses revealed modest relationships between 1-min inter-infusion intervals and progressive-ratio responding and cumulative heroin infusions and responding for heroin when effort is low in behavioral economics (Q0). Even though the correlation found in progressive-ratio did not persist when analyses were separated by access group, the correlation with Q0 persisted uniquely in continuous-access animals, where higher cumulative intake led to higher responding during behavioral economics when effort was low. On the other hand, cumulative heroin infusions for the intermittent-access group led to a moderate to strong negative correlation with alpha (α) values calculated from behavioral economics. In other words, more heroin infusions earned during self-administration is associated with lower α values or sensitivity to demand changes for heroin. Additionally, the particular correlation between 1-min inter-infusion intervals and responding during conditioned reinforcement on abstinence day 7 only emerged in intermittent-access animals while the correlation during this test and cumulative heroin infusions persisted for both access groups. The pattern of these correlations also provides some evidence that pattern of intake and overall intake amount should both be acknowledged as influences on motivation and can be distinguished from each other.

A study by Garcia et al. (2020) examined rats trained to self-administer cocaine on a ContA schedule and found that behavioral measures for terminal intake, initial responding during extinction, and responding during cue-induced reinstatement were dissociable (i.e. uncorrelated). Crucially, although rats escalated intake, not all rats exhibited predictably high responding in the subsequent motivational tests. Separate neurobiological processes may underlie differences in behavioral performance. For instance, suppression of medium spiny neurons (**MSNs**) in the nucleus accumbens projecting to the ventral tegmental area leads to a decrease in conditioned reinforcement of heroin, but does not affect motivation for heroin (progressive-ratio testing), while other MSNs projecting to the ventral pallidum influence motivation, but not conditioned reinforcement (O’Neal et al. 2020). This evidence of separate neurobiological processes independently responsible for intake and motivation complements the results we found where male IntA subjects respond similarly during motivational testing despite lower overall intake in self-administration.

### Conditioned reinforcement

Responding for the heroin-related cue during conditioned reinforcement (i.e. cue-seeking) decreased significantly from abstinence day 7 to day 14. This test differs from traditional models of conditioned reinforcement (Shaham et al. 2003; Shalev et al. 2001) in that there is no extinction of the lever-pressing response, but is similar in that the cue-drug association is not extinguished. Thus, the cue supports responding during the conditioned reinforcement test. Previous studies have found that cue-seeking behavior for opioids increases until 15 days of forced drug abstinence and remains stable for up to 30 days after last heroin exposure (Barrera et al. 2021; Venniro et al. 2019). This difference may be explained in part by the difference in heroin dose used. For example, Barrera et al. (2021) used 0.1 mg/kg infusions of heroin while this study used 0.02 mg/kg per infusion. This lower dose may have affected cue-seeking behavior by altering the withdrawal experience (Dai et al. 1989). The similar decrease in responding from the first to second test could also be due to repeated testing of the same animals. A different method seen in published literature assigned groups of animals to a specific day, then data is examined between subjects rather than within the same group over time (Zhou et al. 2009). We chose to examine conditioned reinforcement within-subjects since HS rats’ behavior tend to be more individually variable due to genetic outbreeding.

We hypothesized there would be an increase in conditioned reinforcement (i.e. cue-seeking) for IntA rats based on findings that IntA is associated with higher (Fragale et al. 2021) or at least comparable responding (Martin et al. 2021) in measures of motivation for other opioids. For example, studies that have compared the difference between short access and long access to heroin have found that longer drug access is associated with escalation of intake, somatic symptoms of withdrawal, and more response during reinstatement tests (Ahmed et al. 2000; Lenoir & Ahmed 2007; Towers et al. 2019). Lenoir and Ahmed (2007) hypothesized that reinstatement of drug-related behaviors could be more dependent on compulsive drug-taking, which only appears to develop with longer self-administration sessions of six hours or more. Further, they considered that responding during reinstatement-type tests could be dissociable from reward sensitization. Our data support this by showing an overall increase in responding during progressive-ratio testing, but not during the conditioned reinforcement test.

The length of time in the operant chamber may be associated with the similar responses in the conditioned reinforcement test because the heroin-related context and cues were still present. Even though the pattern of intake was different in our study, both groups were in the self-administration chambers for four hours. Drugs of abuse including heroin can be subject to behavioral conditioning, where the environment associated with drug use can elicit sensitization or tolerance to certain drug effects. For example, clinicians who interviewed opioid overdose survivors reported that nearly half of the participants had been in an unusual environment when the event occurred (Gerevich et al. 2005; Gutiérrez-Cebollada et al. 1994; Siegel et al. 1982).The significance of environment may explain why the levels of motivation during conditioned reinforcement in our experiment were similar, since the exposure to the drug-associated environment and cues were equal between both drug access groups (IntA vs. ContA). On the other hand, when considering males specifically, ContA animals received significantly more cue associations as a natural consequence to receiving more infusions overall during self-administration, although this did not result in higher responding during the conditioned reinforcement test. Yet, this difference in cue exposure may have counteracted potential group differences in males, which could be addressed in future studies by limiting the number of drug infusions (and thus the drug-cue pairing).

### Sex differences

A limitation of this study is that the effect of sex on progressive-ratio responding could not be determined because several female rats were removed due to catheter failures. This complicates the interpretation of this result by precluding sex-specific conclusions. Females responded similarly to males when collapsed across access group during self-administration and subsequent behavioral testing. This is in contrast with other studies, which have found the females typically have higher opioid intake and responding in motivational tests (Carroll et al. 2002; Cicero et al. 2003; Kimbrough et al. 2020). Due to shorter inter-infusion intervals, female IntA rats were able to exhibit comparable responding during self-administration as male IntA and female ContA counterparts. Despite this comparable responding (or lower when compared to male ContA rats), female IntA rats responded more during behavioral economics for lower doses and showed more willingness to work harder to achieve their desired dose of heroin. Meanwhile, male ContA rats do not work harder than the other *Drug Access* or *Sex* groups despite taking significantly more heroin during self-administration.

This may be related to the heroin dose used. The current study’s heroin dose (20 µg/kg) was similar to some studies (Corre et al. 2018; Doherty et al. 2013; Fattore et al. 2021) but lower than others (~ 50 µg/kg; Ahmed et al. 2000; Bossert et al. 2016; Carmack et al. 2019). The 20 µg/kg heroin dose was chosen based on a study that found lower doses such as 30 µg/kg are sufficient to create strong reinforcement without the need for an infusion cap to avoid overdose and without confounding physical withdrawal symptoms (Dai et al. 1989). Sex differences seen in other studies may thus be more evident with higher, more salient doses. However, more recent studies reported that male and female rats similarly responded for heroin at various doses during self-administration (Barrera et al. 2021; D’Ottavio et al. 2022; Venniro et al. 2019), although these studies did not conduct behavioral economic measures of heroin demand. The lack of differences in these ContA paradigms may also be due to different session lengths as well, because earlier studies tested rats for six rather than four hours (Carroll et al. 2002; D’Ottavio et al. 2022).

The role of sex hormones, particularly estradiol and progesterone, has been studied in relation to susceptibility to drugs of abuse. Roth et al. (2002) concluded that estradiol treatment on ovariectomized female rats allowed for quicker acquisition of heroin-taking and higher heroin consumption overall, which agrees with previous literature concerning psychostimulants. Since we did not measure estrous cycles, it is not known whether this influenced heroin acquisition. Pharmacokinetic research has shown that metabolism of heroin also differs between sexes, where male rats show higher peaks in opiate blood concentrations and opiate brain levels peak later in male rats than female rats (Djurendic-Brenesel et al. 2010; Djurendic-Brenesel et al. 2012). As a result, male rats may perceive the same dose of heroin as more salient than female rats, and this higher perceived salience would influence performance on subsequent motivational tests.

### Considerations & limitations

In addition, the lower intake exhibited by the HS strain of rats may account for the lack of robust differences between groups throughout this study. We chose a lower heroin dose (20 µg/kg per infusion) based on previous studies that used similar doses when conducting progressive-ratio testing and reinstatement tests (Palandri et al. 2021; Smith et al. 2019). Yet, compared to other studies using other rat strains and doses between 40 to 112 µg/kg per infusion (Avvisati et al. 2019; Coffey et al. 2018; D’Ottavio et al. 2022; McNamara et al. 2010), total intake remained low. While these studies demonstrate that intake and motivation are dissociable, the lower intake in our study may have precluded the robust heroin intake required for IntA-induced increases in motivation observed with drugs such as cocaine and fentanyl (e.g., Fragale et al. 2021; Zimmer et al. 2012).

Another consideration that could have affected intake during self-administration is the time-out length for the IntA paradigm. IntA animals received five minutes of drug access every 30 min, which is based on a previous study with cocaine (Zimmer et al. 2012) and corresponds with the paradigm structure in other studies done with heroin (D’Ottavio et al. 2022) and oxycodone (Samson et al. 2022). Since this IntA procedure was originally intended for cocaine that has a half-life of approximately 10 min, it is possible that this time-out period is too short to allow for levels of heroin and its metabolites to clear from the brain by the next drug-accessible period. This clearing of the drug is important to achieve the distinguishable “binge-like” pattern of intake (Zimmer et al. 2012). Heroin has a short half-life (~ 3 min), while its first metabolite 6-monoacetylmorphine (6-MAM) has a half-life of ~ 23 min (Gottas et al. 2012; Rook et al. 2006). 6-MAM is then metabolized into morphine which has an even longer half-life of ~ 50 min (Gottas et al. 2012). Thus, while heroin would clear during the unavailable periods and 6-MAM would approach near-zero levels, morphine would accumulate in the brain throughout the session (D’Ottavio et al. 2022).

To minimize possible unintended effects of morphine accumulation while maintaining the original IntA parameters, the heroin dose was lowered to 20 µg/kg. Despite these precautions taken, we cannot rule out any carry-over effects from these metabolites lasting past the time-out periods. This feature of heroin pharmacokinetics may play a role in the drug-taking habits of humans. In humans, limited cocaine availability can lead to irregular binge-like behavior, during which other risky behaviors are more likely (Beveridge et al. 2012; Cohen and Sas 1994; Roy et al. 2017; Ward et al. 1997). In contrast, human heroin use is characterized by increases in daily intake at predictable intervals, in which withdrawal avoidance may play a contributing role (e.g., Covington and Miczek 2011; Hser et al. 2001). In this sense the animal model of heroin IntA used in this study models this regular binge-like behaviors, although investigation of other time-out durations is warranted.

Lastly, the threshold test conducted to analyze behavioral demand differed from other studies, which limits the interpretation that can be made with the results. Previous researchers have trained animals on this paradigm for up to 14 days (James et al. 2019), when maximum price values between sessions were within three points (Bentzley et al. 2013), or until alpha values were differed less than 25% between sessions (Fragale et al. 2021). This extensive training provides evidence that the animals understand the paradigm and the behaviors analyzed are due to motivation rather than learning. In our experiment, we repeated the paradigm twice, where responding significantly decreased overall during the second iteration. Even though we do not have evidence to prove that this behavior is stable over time due to this decrease in responding on the second instance, we found that the alpha values (which had a significant difference in access group for females) were negatively correlated with progressive-ratio responding (see Fig. 9). This moderate correlation gives some confidence that responding during the threshold test is not purely from learning the paradigm. However, future studies that we conduct that utilize this behavioral economics procedure should focus on replicating and extending these findings after longer training to verify that any of these differences are stable and due to motivation without any confounding from the initial learning.

### Implications

It is important to understand how OUD develops and the factors influence its development, so that behavioral and pharmacological treatments can be improved. This study demonstrates that the pattern of drug intake can influence the trajectory of drug motivation. We found that IntA to heroin induces incentive sensitization as measured by the behavioral economics test of heroin demand and created similar cue-seeking behavior in male rats despite less overall intake during self-administration. These results suggest that IntA self-administration allows for the dissociation of intake from motivation, particularly observed by similar responding in progressive-ratio and lower α values in behavioral economics despite having lower overall intake, especially in male rats.

Further research is needed to distinguish the neurobiological effects of heroin intake under IntA compared to ContA paradigms. Previous researchers have concluded that opioids such as heroin activate more brain areas than other drugs of abuse like cocaine and have more effects in brain areas important for maintenance of drug-seeking. For example, cocaine use appears to increase dopamine levels in the NAc while heroin use indirectly increases dopamine in the accumbens by blocking ventral tegmental inhibitory neurons (Badiani et al. 2011). Only approximately 20% of NAc neurons are activated by both heroin and cocaine (Chang et al.^1998^). Moreover, opioids like morphine can have the opposite long-term effect as psychostimulants in the NAc, decreasing dendritic density of medium-spiny neurons (Lefevre et al. 2023; Robinson et al. 2002; Siemsen et al. 2023). Since opioids such as heroin work so differently from other drugs of abuse that have been studied, future research should focus on illuminating the neurobiological changes that occur from long-term opioid use. This research objective will help improve pharmacological treatments for OUD and overall increase the understanding of this unique disorder.

In conclusion, these data support that the pattern of heroin intake is a contributing factor to the sensitization of heroin motivation. Specifically, IntA led to similar responding during motivational testing despite less intake during self-administration for males while IntA resulted in less demand elasticity during behavioral economics with similar intake for females. This discovery helps improve preclinical animal models of addiction and provide insight into how pattern of intake may affect the trajectory of drug use in humans. IntA models demonstrate human drug-taking more accurately, where drug is taken in small periods of time with breaks between use to allow for other activities. Further, females did not exhibit the same results as male rats, indicating the importance of testing both sexes to discern the differences in self-administration and motivation of heroin.

## Data Availability

The data that support the findings of this study are available from the corresponding author upon request.

## References

[CR1] Ahmed SH, Walker JR, Koob GF (2000) Persistent increase in the motivation to take heroin in rats with a history of drug escalation. Neuropsychopharmacology 22(4):413–421. 10.1016/s0893-133x(99)00133-5 10700660 10.1016/S0893-133X(99)00133-5

[CR2] Allain F, Bouayad-Gervais K, Samaha AN (2018) High and escalating levels of cocaine intake are dissociable from subsequent incentive motivation for the drug in rats. Psychopharmacology 235(1):317–328. 10.1007/s00213-017-4773-8 29085961 10.1007/s00213-017-4773-8

[CR3] Allain F, Samaha AN (2019) Revisiting long-access versus short-access cocaine self-administration in rats: intermittent intake promotes addiction symptoms independent of session length. Addict Biol 24(4):641–651. 10.1111/adb.12629 29920865 10.1111/adb.12629

[CR4] American Psychiatric Association (2013) Diagnostic and statistical manual of mental disorders (5th ed). 10.1176/appi.books.9780890425596

[CR5] Avvisati R, Bogen IL, Andersen JM, Vindenes V, Mørland J, Badiani A, Boix F (2019) The active heroin metabolite 6-acetylmorphine has robust reinforcing effects as assessed by self-administration in the rat. Neuropharmacology 15(150):192–199. 10.1016/j.neuropharm.2018.12.023 10.1016/j.neuropharm.2018.12.023 30578794

[CR6] Badiani A, Belin D, Epstein D, Calu D, Shaham Y (2011) Opiate versus psychostimulant addiction: the differences do matter. Nat Rev Neurosci 12(11):685–700. 10.1038/nrn3104 21971065 10.1038/nrn3104 PMC3721140

[CR7] Barrera ED, Loughlin L, Greenberger S, Ewing S, Hachimine P, Ranaldi R (2021) Environmental enrichment reduces heroin seeking following incubation of craving in both male and female rats. Drug Alcohol Depend 226:108852. 10.1016/j.drugalcdep.2021.108852 34225225 10.1016/j.drugalcdep.2021.108852 PMC8355213

[CR8] Beasley MM, Tunstall BJ, Kearns DN (2023) Intermittent access cocaine self-administration produces context-specific escalation and increased motivation. Drug Alcohol Depend 1(245):109797. 10.1016/j.drugalcdep.2023 10.1016/j.drugalcdep.2023.109797 PMC10033440 36801708

[CR9] Bentzley BS, Fender KM, Aston-Jones G (2013) The behavioral economics of drug self-administration: a review and new analytical approach for within-session procedures. Psychopharmacology 226(1):113–125. 10.1007/s00213-012-2899-2 23086021 10.1007/s00213-012-2899-2 PMC3572328

[CR10] Bentzley BS, Jhou TC, Aston-Jones G (2014) Economic demand predicts addiction-like behavior and therapeutic efficacy of oxytocin in the rat. Proc Natl Acad Sci U S A 111(32):11822–11827. 10.1073/pnas.1406324111 25071176 10.1073/pnas.1406324111 PMC4136574

[CR11] Beveridge T, Wray P, Brewer A (2012) Analyzing human cocaine use patterns to inform animal addiction model development. Paper presented at the College of Problems of Drug Dependence Annual Meeting, Palm Springs, CA

[CR12] Bossert JM, Adhikary S, St Laurent R, Marchant NJ, Wang HL, Morales M, Shaham Y (2016) Role of projections from ventral subiculum to nucleus accumbens shell in context-induced reinstatement of heroin seeking in rats. Psychopharmacology 233(10):1991–2004. 10.1007/s00213-015-4060-5 26344108 10.1007/s00213-015-4060-5 PMC4781679

[CR13] Bouayad-Gervais K, Minogianis EA, Lévesque D, Samaha AN (2014) The self-administration of rapidly delivered cocaine promotes increased motivation to take the drug: contributions of prior levels of operant responding and cocaine intake. Psychopharmacology 231(21):4241–4252. 10.1007/s00213-014-3576-4 24752656 10.1007/s00213-014-3576-4

[CR14] Carmack SA, Keeley RJ, Vendruscolo JCM, Lowery-Gionta EG, Lu H, Koob GF, Vendruscolo LF (2019) Heroin addiction engages negative emotional learning brain circuits in rats. J Clin Investig 129(6):2480–2484. 10.1172/JCI125534 30913040 10.1172/JCI125534 PMC6546476

[CR15] Carroll ME, Morgan AD, Lynch WJ, Campbell UC, Dess NK (2002) Intravenous cocaine and heroin self-administration in rats selectively bred for differential saccharin intake: phenotype and sex differences. Psychopharmacology (Berl) 161(3):304–313. 10.1007/s00213-002-1030-5 12021834 10.1007/s00213-002-1030-5

[CR16] Centers for Disease Control and Prevention (2022) Heroin. Retrieved from https://www.cdc.gov/opioids/basics/heroin.html. Accessed June 2024

[CR17] Chang JY, Janak PH, Woodward DJ (1998) Comparison of mesocorticolimbic neuronal responses during cocaine and heroin self-administration in freely moving rats. J Neurosci 18(8):3098–3115. 10.1523/JNEUROSCI.18-08-03098.1998 9526026 10.1523/JNEUROSCI.18-08-03098.1998 PMC6792596

[CR18] Chen SA, O’Dell LE, Hoefer ME, Greenwell TN, Zorrilla EP, Koob GF (2006) Unlimited access to heroin self-administration: independent motivational markers of opiate dependence. Neuropsychopharmacology 31(12):2692–2707. 10.1038/sj.npp.1301008 16452993 10.1038/sj.npp.1301008

[CR19] Cicero TJ, Aylward SC, Meyer ER (2003) Gender differences in the intravenous self-administration of mu opiate agonists. Pharmacol Biochem Behav 74(3):541–549. 10.1016/S0091-3057(02)01039-0 12543217 10.1016/s0091-3057(02)01039-0

[CR20] Coffey AA, Fang J, Grigson PS (2018) Heroin self-administration as a function of time of day in rats. Psychopharmacology (Berl) 235(10):3005–3015. 10.1007/s00213-018-4990-9 30178302 10.1007/s00213-018-4990-9 PMC6162178

[CR21] Cohen P, Sas A (1994) Cocaine use in Amsterdam in non deviant subcultures. Addict Res 2:71–94. 10.3109/16066359409005547

[CR22] Corre J, van Zessen R, Loureiro M, Patriarchi T, Tian L, Pascoli V, Lüscher C (2018) Dopamine neurons projecting to medial shell of the nucleus accumbens drive heroin reinforcement. Elife 7:e39945. 10.7554/eLife.39945 30373717 10.7554/eLife.39945 PMC6207421

[CR23] Covington HE, Miczek KA (2011) Binge drug taking. In: Olmstead M (eds) Animal models of drug addiction. Neuromethods, vol 53. Humana. 10.1007/978-1-60761-934-5_15

[CR24] D’Ottavio G, Reverte I, Ragozzino D, Meringolo M, Milella MS, Boix F, Caprioli D (2022) Increased heroin intake and relapse vulnerability in intermittent relative to continuous self-administration: sex differences in rats. British journal of pharmacology. 10.1111/bph.15791 34986504 10.1111/bph.15791 PMC9253203

[CR25] Dai S, Corrigall WA, Coen KM, Kalant H (1989) Heroin self-administration by rats: influence of dose and physical dependence. Pharmacol Biochem Behav 32(4):1009–1015. 10.1016/0091-3057(89)90074-9 2798525 10.1016/0091-3057(89)90074-9

[CR26] Djurendic-Brenesel M, Mimica-Dukic N, Pilija V, Tasic M (2010) Gender-related differences in the pharmacokinetics of opiates. Forensic Sci Int 194(1–3):28–33. 10.1016/j.forsciint.2009.10.003 19913374 10.1016/j.forsciint.2009.10.003

[CR27] Djurendic-Brenesel M, Pilija V, Mimica-Dukic N, Budakov B, Cvjeticanin S (2012) Distribution of opiate alkaloids in brain tissue of experimental animals. Interdiscip Toxicol 5(4):173–178. 10.2478/v10102-012-0029-y 23554560 10.2478/v10102-012-0029-y PMC3600520

[CR28] Doherty JM, Cooke BM, Frantz KJ (2013) A role for the prefrontal cortex in heroin-seeking after forced abstinence by adult male rats but not adolescents. Neuropsychopharmacology 38(3):446–454. 10.1038/npp.2012.200 23072838 10.1038/npp.2012.200 PMC3547195

[CR29] Fattore L, Fadda P, Zanda MT, Fratta W (2021) Analysis of opioid-seeking behavior through the intravenous self-administration reinstatement model in rats. Methods Mol Biol 2201:231–245. 10.1007/978-1-0716-0884-5_21 32975804 10.1007/978-1-0716-0884-5_21

[CR30] Fragale JE, James MH, Aston-Jones G (2021) Intermittent self-administration of fentanyl induces a multifaceted addiction state associated with persistent changes in the orexin system. Addict Biol 26(3):e12946. 10.1111/adb.12946 32798290 10.1111/adb.12946 PMC7882007

[CR31] Garcia AF, Webb IG, Yager LM, Seo MB, Ferguson SM (2020) Intermittent but not continuous access to cocaine produces individual variability in addiction susceptibility in rats. Psychopharmacology 237(10):2929–2941. 10.1007/s00213-020-05581-1 32556402 10.1007/s00213-020-05581-1 PMC7529862

[CR32] Gerevich J, Bácskai E, Farkas L, Danics Z (2005) A case report: pavlovian conditioning as a risk factor of heroin “overdose” death. Harm Reduct J 2:11. 10.1186/1477-7517-2-11 16042795 10.1186/1477-7517-2-11 PMC1196296

[CR33] Gottas A, Oiestad EL, Boix F, Ripel A, Thaulow CH, Pettersen BS, Vindenes V, Morland J (2012) Simultaneous measurement of heroin and its metabolites in brain extracellular fluid by microdialysis and ultra performance liquid chromatography tandem mass spectrometry. J Pharmacol Toxicol Methods 66(1):14–21. 10.1016/j.vascn.2012.04.009 22561414 10.1016/j.vascn.2012.04.009

[CR34] Gutiérrez-Cebollada J, de la Torre R, Ortuño J, Garcés JM, Camí J (1994) Psychotropic drug consumption and other factors associated with heroin overdose. Drug Alcohol Depend 35(2):169–174. 10.1016/0376-8716(94)90124-4 7914483 10.1016/0376-8716(94)90124-4

[CR35] Hedegaard H, Miniño AM, Spencer MR, Warner M (2021) Drug overdose deaths in the United States, 1999–2020. Retrieved from https://stacks.cdc.gov/view/cdc/112340. Accessed June 2024 34978529

[CR36] Hser Y, Hoffman V, Grella CE, Anglin MD (2001) A 33-year follow-up of narcotics addicts. Arch Gen Psychiatry 58(5):503–508. 10.1001/archpsyc.58.5.503 11343531 10.1001/archpsyc.58.5.503

[CR37] Hursh SR, Silberberg A (2008) Economic demand and essential value. Psychol Rev 115(1):186–198. 10.1037/0033-295x.115.1.186 18211190 10.1037/0033-295X.115.1.186

[CR38] James MH, Stopper CM, Zimmer BA, Koll NE, Bowrey HE, Aston-Jones G (2019) Increased number and activity of a lateral subpopulation of hypothalamic orexin/hypocretin neurons underlies the expression of an addicted state in rats. Biol Psychiatry 85(11):925–935. 10.1016/j.biopsych.2018.07.022 30219208 10.1016/j.biopsych.2018.07.022 PMC7528037

[CR39] Kawa AB, Bentzley BS, Robinson TE (2016) Less is more: prolonged intermittent access cocaine self-administration produces incentive-sensitization and addiction-like behavior. Psychopharmacology 233(19–20):3587–3602. 10.1007/s00213-016-4393-8 27481050 10.1007/s00213-016-4393-8 PMC5023484

[CR40] Kenny PJ, Chen SA, Kitamura O, Markou A, Koob GF (2006) Conditioned withdrawal drives heroin consumption and decreases reward sensitivity. J Neurosci 26(22):5894–5900. 10.1523/jneurosci.0740-06.2006 16738231 10.1523/JNEUROSCI.0740-06.2006 PMC6675212

[CR41] Kimbrough A, Kononoff J, Simpson S, Kallupi M, Sedighim S, Palomino K, George O (2020) Oxycodone self-administration and withdrawal behaviors in male and female Wistar rats. Psychopharmacology 237(5):1545–1555. 10.1007/s00213-020-05479-y 32114633 10.1007/s00213-020-05479-y PMC7269712

[CR42] Lefevre EM, Gauthier EA, Bystrom LL, Scheunemann J, Rothwell PE (2023) Differential patterns of synaptic plasticity in the nucleus accumbens caused by continuous and interrupted morphine exposure. J Neurosci 43(2):308–318. 10.1523/JNEUROSCI.0595-22.2022 36396404 10.1523/JNEUROSCI.0595-22.2022 PMC9838694

[CR43] Lenoir M, Ahmed SH (2007) Heroin-induced reinstatement is specific to compulsive heroin use and dissociable from heroin reward and sensitization. Neuropsychopharmacology 32(3):616–624. 10.1038/sj.npp.1301083 16641938 10.1038/sj.npp.1301083

[CR44] Martin DA, Gyawali U, Calu DJ (2021) Effects of 5-HT(2A) receptor stimulation on economic demand for fentanyl after intermittent and continuous access self-administration in male rats. Addict Biol 26(3):e12926. 10.1111/adb.12926 32458577 10.1111/adb.12926 PMC7688480

[CR45] McNamara R, Dalley JW, Robbins TW, Everitt BJ, Belin D (2010) Trait-like impulsivity does not predict escalation of heroin self-administration in the rat. Psychopharmacology 212(4):453–464. 10.1007/s00213-010-1974-9 20689939 10.1007/s00213-010-1974-9

[CR46] Minhas M, Leri F (2014) The effect of heroin dependence on resumption of heroin self-administration in rats. Drug Alcohol Depend 138:24–31. 10.1016/j.drugalcdep.2014.01.007 24613630 10.1016/j.drugalcdep.2014.01.007

[CR47] National Research Council (US) Committee for the Update of the Guide for the Care and Use of Laboratory Animals (2011) Guide for the care and use of laboratory animals: Eighth edition. Washington, DC: The National Academies Press. 10.17226/12910 21595115

[CR48] Newman M, Ferrario CR (2020) An improved demand curve for analysis of food or drug consumption in behavioral experiments. Psychopharmacology 237(4):943–955. 10.1007/s00213-020-05491-2 32170328 10.1007/s00213-020-05491-2 PMC7113227

[CR49] O’Neal TJ, Nooney MN, Thien K, Ferguson SM (2020) Chemogenetic modulation of accumbens direct or indirect pathways bidirectionally alters reinstatement of heroin-seeking in high- but not low-risk rats. Neuropsychopharmacology 45(8):1251–1262. 10.1038/s41386-019-0571-9 31747681 10.1038/s41386-019-0571-9 PMC7297977

[CR50] Palandri J, Smith SL, Heal DJ, Wonnacott S, Bailey CP (2021) Contrasting effects of the α7 nicotinic receptor antagonist methyllycaconitine in different rat models of heroin reinstatement. J Psychopharmacol 35(10):1204–1215. 10.1177/0269881121991570 33691518 10.1177/0269881121991570 PMC8521373

[CR51] Richardson NR, Roberts DC (1996) Progressive ratio schedules in drug self-administration studies in rats: a method to evaluate reinforcing efficacy. J Neurosci Methods 66(1):1–11. 10.1016/0165-0270(95)00153-0 10.1016/0165-0270(95)00153-0 8794935

[CR52] Robinson TE, Berridge KC (1993) The neural basis of drug craving: an incentive-sensitization theory of addiction. Brain Res Rev 18(3):247–291. 10.1016/0165-0173(93)90013-p 8401595 10.1016/0165-0173(93)90013-p

[CR53] Robinson TE, Gorny G, Savage VR, Kolb B (2002) Widespread but regionally specific effects of experimenter- versus self-administered morphine on dendritic spines in the nucleus accumbens, hippocampus, and neocortex of adult rats. Synapse 46(4):271–279. 10.1002/syn.10146 12373743 10.1002/syn.10146

[CR54] Rook EJ, Huitema AD, van den Brink W, van Ree JM, Beijnen JH (2006) Pharmacokinetics and pharmacokinetic variability of heroin and its metabolites: review of the literature. Curr Clin Pharmacol 1(1):109–118. 10.2174/157488406775268219 18666382 10.2174/157488406775268219

[CR55] Roth ME, Casimir AG, Carroll ME (2002) Influence of estrogen in the acquisition of intravenously self-administered heroin in female rats. Pharmacol Biochem Behav 72(1):313–318. 10.1016/S0091-3057(01)00777-8 11900802 10.1016/s0091-3057(01)00777-8

[CR56] Roy É, Arruda N, Jutras-Aswad D, Berbiche D, Perreault M, Bertrand K, Dufour M, Bruneau J (2017) Examining the link between cocaine binging and individual, social and behavioral factors among street-based cocaine users. Addict Behav 68:66–72. 10.1016/j.addbeh.2017.01.012 28103534 10.1016/j.addbeh.2017.01.012

[CR57] Samson KR, Xu W, Kortagere S, España RA (2022) Intermittent access to oxycodone decreases dopamine uptake in the nucleus accumbens core during abstinence. Addict Biol 27(6):e13241. 10.1111/adb.13241 36301217 10.1111/adb.13241 PMC10262085

[CR58] Shaham Y, Shalev U, Lu L, de Wit H, Stewart J (2003) The reinstatement model of drug relapse: history, methodology, and major findings. Psychopharmacology 168:3–20. 10.1007/s00213-002-1224-x 12402102 10.1007/s00213-002-1224-x

[CR59] Shalev U, Morales M, Hope B, Yap J, Shaham Y (2001) Time-dependent changes in extinction behavior and stress-induced reinstatement of drug seeking following withdrawal from heroin in rats. Psychopharmacology 156(1):98–107. 10.1007/s002130100748 11465640 10.1007/s002130100748

[CR60] Siegel S, Hinson RE, Krank MD, McCully J (1982) Heroin “overdose” death: contribution of drug-associated environmental cues. Science 216(4544):436–437. 10.1126/science.7200260 7200260 10.1126/science.7200260

[CR61] Siemsen BM, Denton AR, Parrila-Carrero J, Hooker KN, Carpenter EA, Prescot ME, Scofield MD (2023) Heroin self-administration and extinction increase prelimbic cortical astrocyte-synapse proximity and alter dendritic spine morphometrics that are reversed by N-acetylcysteine. Cells 12(14):1812. 10.3390/cells12141812 37508477 10.3390/cells12141812 PMC10378353

[CR62] Smith SL, Dean RL, Todtenkopf MS, Heal DJ (2019) Investigation of the reinforcing potential of samidorphan and naltrexone by fixed and progressive ratio intravenous self-administration testing in heroin-maintained rats. J Psychopharmacol 33(3):383–391. 10.1177/0269881118822111 30676189 10.1177/0269881118822111

[CR63] Solberg Woods LC, Stelloh C, Regner KR, Schwabe T, Eisenhauer J, Garrett MR (2010) Heterogeneous stock rats: a new model to study the genetics of renal phenotypes. Am J Physiol Renal Physiol 298(6):F1484–1491. 10.1152/ajprenal.00002.2010 20219828 10.1152/ajprenal.00002.2010 PMC2886820

[CR64] Substance Abuse and Mental Health Services Administration (2022) Key substance use and mental health indicators in the United States: results from the 2021 National Survey on Drug Use and Health (HHS Publication No. PEP22-07-01-005, NSDUH Series H-57). Center for Behavioral Health Statistics and Quality, Substance Abuse and Mental Health Services Administration. https://www.samhsa.gov/data/report/2021-nsduh-annual-national-report. Accessed Mar 2024

[CR65] Towers EB, Tunstall BJ, McCracken ML, Vendruscolo LF, Koob GF (2019) Male and female mice develop escalation of heroin intake and dependence following extended access. Neuropharmacology 151:189–194. 10.1016/j.neuropharm.2019.03.019 30880124 10.1016/j.neuropharm.2019.03.019 PMC9345532

[CR66] Venniro M, Russell TI, Zhang M, Shaham Y (2019) Operant social reward decreases incubation of heroin craving in male and female rats. Biol Psychiat 86(11):848–856. 10.1016/j.biopsych.2019.05.018 31326085 10.1016/j.biopsych.2019.05.018 PMC8383184

[CR67] Wang L, Volkow ND, Berger NA, Davis PB, Kaelber DC, Xu R (2023) Association of COVID-19 with endocarditis in patients with cocaine or opioid use disorders in the US. Mol Psychiatry 28(2):543–552. 10.1038/s41380-022-01903-1 36510003 10.1038/s41380-022-01903-1 PMC9918660

[CR68] Ward AS, Haney M, Fischman MW, Foltin RW (1997) Binge cocaine self-administration in humans: intravenous cocaine. Psychopharmacology (Berl) 132(4):375–81. 10.1007/s002130050358 10.1007/s002130050358 9298515

[CR69] Zhang F, Zhou W, Tang S, Lai M, Liu H, Yang G (2004) Motivation of heroin-seeking elicited by drug-associated cues is related to total amount of heroin exposure during self-administration in rats. Pharmacol Biochem Behav 79(2):291–298. 10.1016/j.pbb.2004.08.001 15501304 10.1016/j.pbb.2004.08.001

[CR70] Zhou W, Zhang F, Liu H, Tang S, Lai M, Zhu H, Kalivas P (2009) Effects of training and withdrawal periods on heroin seeking induced by conditioned cue in an animal of model of relapse. Psychopharmacology 203(4):677–684. 10.1007/s00213-008-1414-2 10.1007/s00213-008-1414-2 19043694

[CR71] Zimmer BA, Oleson EB, Roberts DC (2012) The motivation to self-administer is increased after a history of spiking brain levels of cocaine. Neuropsychopharmacology 37(8):1901–1910. 10.1038/npp.2012.37 22453139 10.1038/npp.2012.37 PMC3376322

